# “Ideal” parathyroid hormone in erythropoietin‐stimulating agents‐resistant anemia

**DOI:** 10.1002/jha2.316

**Published:** 2021-11-24

**Authors:** Bilal Ashraf, Taha Bat, Olga K. Weinberg, Orson W. Moe, Ibrahim Ibrahim

**Affiliations:** ^1^ Division of Hematology and Oncology Department of Internal Medicine University of Texas Southwestern Medical Center Dallas Texas USA; ^2^ Department of Pathology Hematopathology Section University of Texas Southwestern Medical Center Dallas Texas USA; ^3^ Division of Nephrology Department of Internal Medicine University of Texas Southwestern Medical Center Dallas Texas USA; ^4^ Charles and Jane Pak Center for Mineral Metabolism and Clinical Research University of Texas Southwestern Medical Center Dallas Texas USA

**Keywords:** bone marrow fibrosis, ESA‐resistant anemia, ESRD, PTH, secondary hyperparathyroidism

## Abstract

Erythropoietin‐stimulating agents (ESAs) have revolutionized anemia treatment in end‐stage renal disease (ESRD), but ESA resistance is increasingly identified. Secondary hyperparathyroidism (SHP) is one cause of ESA resistance. We describe a patient with ESA‐resistant, transfusion‐dependent anemia and mild SHP with remodeling and reticulin fibrosis on bone marrow biopsy, all of which resolved with stricter SHP management. We identified 64 patients with anemia, ESRD, and bone marrow biopsy. The parathyroid hormone (PTH) range for bony remodeling was 183–16,161.9 pg/ml versus 90.8–3283 pg/ml. The PTH range for fibrotic changes was 183–2487 pg/ml versus 90.8–16,161.9 pg/ml. We found no clear PTH range predictive for bone marrow changes.

## 1 INTRODUCTION

Both anemia and secondary hyperparathyroidism (SHP) are common complications of chronic kidney disease (CKD) and end‐stage renal disease (ESRD). Anemia in ESRD is most often attributed to decreased relative erythropoietin (EPO) secretion from the kidney [1]. As a consequence, treatment is centered on iron repletion and erythropoietin‐stimulating‐agent (ESA) administration [2]. However, in 10% of cases of renal anemia, resistance to ESAs is notable: a phenomenon known as ESA‐resistant anemia [3, 4]. Factors implicated in ESA‐resistant anemia include inadequate dialysis, SHP, thyroid dysfunction, vitamin D deficiency, and myelodysplasia [3]. Poorly controlled SHP, indicated by elevated parathyroid hormone (PTH) levels, is often associated with severe anemia. However, the ideal PTH level in ESA‐resistant anemia has not been determined and likely vary from subject to subject.

In this paper, we describe a patient with ESRD, SHP and associated bone marrow changes in the setting of transfusion‐dependent anemia. With treatment of SHP, the bone marrow findings and transfusion dependence is completely reversed. In addition, we share descriptive findings of bone marrow biopsies in patients with anemia and ESRD in our center.

## 2 CASE PRESENTATION

**FIGURE 1:**
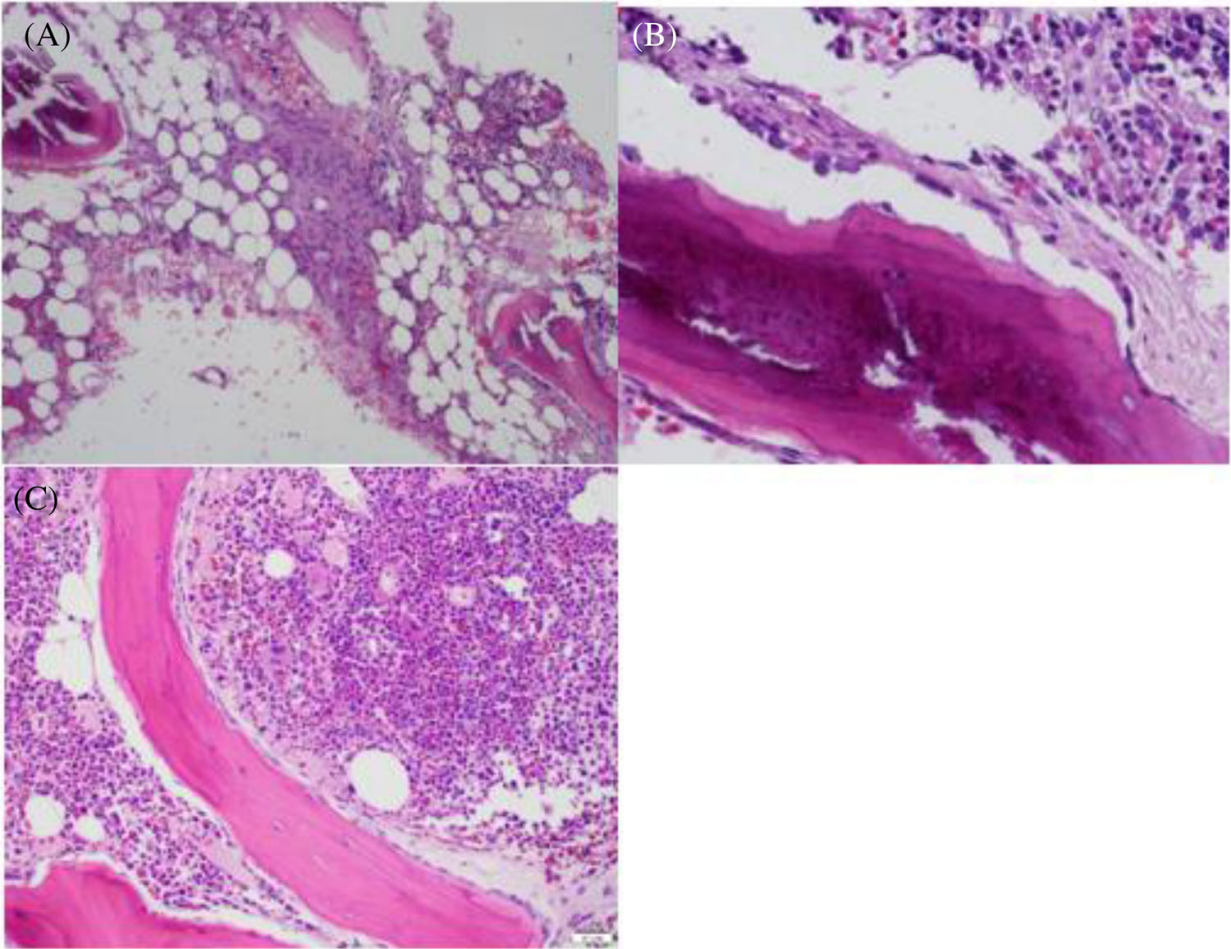
Bone marrow biopsy findings prior to treatment of Secondary hyperparathyroidism (SHP) revealed fibrotic changes (A) and bony remodeling (B). Bone marrow biopsy findings after treatment of SHP no longer revealed fibrotic changes or bony remodeling (C)

A 68‐year‐old male with a history of ESRD secondary to type 2 diabetes and hypertension was referred to our hematology clinic for transfusion‐dependent ESA‐resistant anemia. He started on peritoneal dialysis 1 year prior and was adherent to dialysis. He was maintained on epoetin 20,000 units three times weekly, but required regular transfusions on a monthly basis over the prior year. Labwork was remarkable for a hemoglobin of 5.3 g/dl with an mean corpuscular volume (MCV) of 103.1. WBC and platelet count were normal. Iron studies revealed a ferritin of 414 ng/ml, iron 184 mcg/dl, TIBC 199 mcg/dl, and iron sat 92%. Reticulocyte percent was 3.0%. Folate, B12 and TSH were within normal limits. Chromosome analysis showed no abnormal clones and was 46 X,Y. He had undergone extensive workup including upper endoscopy, colonoscopy as well as capsule endoscopy without any clear source of anemia. Bone marrow biopsy was performed and revealed a mostly normocellular bone marrow at 30%–40% with focal alternating areas of hypercellularity. It further revealed trilineage hematopoiesis with mild reticulin fibrosis and focal areas of bony remodeling (findings that were confirmed in our institutional pathology review) (Figure 1A).

Our clinical evaluation revealed a normal SPEP, kappa/lambda free light chain ratio, and a mildly low copper at 0.57 mcg/ml (0.85–1.8). PTH was modestly elevated at 400.5 pg/ml (10–55). Upon discussion with the patient's primary nephrologist, we recommended optimization of his SHP given his initial bone marrow findings with bony remodeling. His prior regimen consisted of calcitriol 0.25 mcg once daily and calcium acetate 667 mg three times daily. Following our consultation, he started on sevelamer 1600 mg three times daily and cinacalcet 30 mg twice daily, while calcium acetate was stopped. The patient was able to maintain a hemoglobin between 7.4 and 9.1 g/dl over the next several months without transfusion, along with an MCV of 97.8. He did require transfusion during an acute infection with SARS‐CoV2, but again became transfusion independent following recovery. A second repeat bone marrow biopsy after 5 months of SHP optimization revealed a hypercellular bone marrow with 70% cellularity and normal trilineage hematopoiesis with no evidence of reticulin fibrosis or bony remodeling (Figure 1B,C).

## 3 RETROSPECTIVE CHART REVIEW

The study was determined to be exempt by the institutional review board at our institution. The electronic medical record was queried for 64 patients with ESRD, anemia, and bone marrow biopsy performed between December 2010 and October 2020 and reviewed at our institution. Charts were individually reviewed for demographic factors, dialysis history (e.g., adherence, modality), and bone marrow biopsy morphological findings.

The results of our chart review are presented in Table 1. The range of PTH for patients with bony remodeling was 183–16,161.9 pg/ml versus 90.8–3283 pg/ml for those without bony remodeling. The range of PTH for patients with fibrotic changes was 183–2487 pg/ml versus 90.8–16,161.9 pg/ml for those without fibrotic changes. The range of hemoglobin for patients with bony remodeling was 6.6–9.8 g/dl versus 6.8–9.6 g/dl for those without bony remodeling. The range of hemoglobin for patients with fibrotic changes was 7–8.4 g/dl versus 6.6‐9.8 g/dl for those without fibrotic changes.

**TABLE 1:** A summary of demographics, lab values, Erythropoietin‐stimulating agent (ESA) dosing and bone marrow findings of all patients with anemia, end‐stage renal disease (ESRD) and a bone marrow biopsy performed at our institution between 2010 and 2020

**Age**	**Gender**	**Race/ethnicity**	**PTH (pg/ml)**	**Hemoglobin (g/dl)**	**ESA dosing**	**Dialysis modality**	**Bone marrow fibrosis**	**Bony remodeling**
32	Female	African American	2763	7.2	Darbopoetin 40 mcg weekly	HD	Marked fibrosis	Yes
13	Female	African American	16161.9	6.6	Epoetin 10,000 units three times weekly	HD	No	Yes
13	Female	African American	2487	7.0	Epoetin 10,000 units three times weekly	HD	Marked fibrosis	Yes
36	Male	Unknown	183	8.4	Epoetin 20,000 units weekly	PD	1–2 + reticulin fibrosis	Yes
54	Female	African American	500	9.8	Darbopoetin 75 mcg weekly	HD	No	Yes
20	Male	White hispanic	3283	8.6	Epoetin 30,000 units weekly	PD	No	Yes
53	Female	Other hispanic	207.8	8.6	Darbopoetin 100 mcg weekly	HD	No	No
20	Female	African American	246.7	8.2	Darbopoetin 60 mcg weekly	HD	No	No
47	Female	White hispanic	156.4	8.1	Epoetin 45,000 units weekly	HD	No	No
74	Male	Caucasian	121	9.2	Darbopoetin 60 mcg weekly	HD	No	No
52	Male	African American	116.8	8.9	Darbopoetin 60 mcg weekly	HD	No	No
33	Female	White Hispanic	183.2	8.3	Darbopoetin 25 mcg weekly	HD	No	No
63	Male	African American	599	8.3	Darbopoetin 60 mcg weekly	HD	No	No
29	Male	Other Hispanic	90.8	7.5	Epoetin 10,000 units weekly	PD	No	No
70	Female	African American	777.7	9.4	Epoetin 40,000 units weekly	HD	No	No
37	Male	African American	340.4	8.6	Epoetin 15,000 units weekly	HD	No	No
54	Male	Asian	329.5	8.1	Darbopoetin 100 mcg weekly	HD	No	No
45	Male	White Hispanic	452.4	6.8	Dose unknown	HD	No	No
57	Female	African American	127.3	8	Epoetin 6000 units weekly	HD	No	No
56	Male	Caucasian	76.8	7.3	Darbopoetin 60 mcg weekly	HD	No	No
56	Male	Caucasian	504.7	8.4	Darbopoetin 40 mcg weekly	HD	No	No
54	Female	White Hispanic	171.9	6.7	Dose unknown	HD	No	No
82	Male	White Hispanic	162.8	8.3	Dose unknown	HD	No	No
67	Female	African American	437.6	9.6	Darbopoetin 40 mcg weekly	HD	No	No
64	Male	African American	228.3	6.6	Epoetin 15,000 units weekly	HD	No	No

## 4 DISCUSSION

SHP is an increasingly defined cause of ESA‐resistant anemia in patients with ESRD [3]. We describe a patient with a modest elevation in PTH who had reversible transfusion‐dependent anemia in the setting of reversible bone marrow changes when management of his SHP was optimized. The severe degree of anemia and transfusion dependence, despite a PTH of only 400.5 pg/ml, and the documented reversibility of the bone marrow fibrosis and achievement of transfusion independence with improvement of PTH levels highlight an important novel observation. Furthermore, the findings from our review of all bone marrow biopsies for patients with anemia and ESRD over a 10‐year period at our institution reveal a greatly variable range of PTH levels at which patients display bone marrow changes. The great variability in PTH levels of both affected and unaffected patients (i.e., bone marrow fibrosis and/or remodeling) indicates the relationship between SHP and ESA‐resistant anemia is complex and likely unique for each patient.

The relationship between SHP and anemia has long been described in the realm of parathyroidectomy. Zingraff et al. [5] first described the phenomenon of increased mean hematocrit following parathyroidectomy in a small series of patients with ESRD. One potential hypothesis suggests that elevated PTH contributes to a shorter erythrocyte life span. Wu et al. [6] demonstrated that ESRD patients with higher levels of PTH had increased osmotic fragility, thus decreasing erythrocyte life span and worsening anemia. What appears to be more relevant in the setting of our patient is the changes associated with SHP at the level of the bone and marrow. One potential mechanism for this relationship, seen in our patient, is increased bone turnover in more severe SHP. Rao et al. [7] showed that anemic ESRD patients who had a poor response to EPO agents had higher levels of PTH and had higher percentages of osteoclastic, eroded bone surfaces. A second suggested mechanism for anemia in SHP is fibrosis, which was also manifested in our patient as reticulin fibrosis. Brickmann et al. [8] demonstrated that elevated PTH levels in patients with ESRD appear to induce fibrosis in the bone marrow. The above study by Rao et al. further showed that ESRD patients with poor EPO response had increased degrees of bone marrow fibrosis [7].

Irrespective of the mechanism by which SHP contributes to ESA‐resistant anemia, it appears that intervention improves anemia, as was seen in our patient. Historically, Barbour et al. [9] described a phenomenon in which patients with ESRD and SHP had increased hematocrit following parathyroidectomy. In the following decades, subsequent studies have demonstrated that medical management of SHP may improve anemia in ESRD. In SHP, both calcitriol and cinacalcet have been shown to increase hematocrit and decrease ESA dose [10, 11, 12], similar to our patient who became transfusion independent following cinacalcet administration.

While the literature does seem to indicate that there is a strong relationship between SHP and ESA‐resistant anemia, treatment of SHP is primarily dictated by goals in PTH, vitamin D, calcium, and phosphorus levels; even these goals that dictate clinical practice are not always scientifically based[10]. No guidelines currently exist on PTH goals in the treatment of SHP when targeting improvement in ESA‐resistant anemia. As described earlier with our patient and our single‐center data, there appears to be great variability in the relationship between PTH level and both anemia and bone marrow changes. One ideal PTH level for the entire dialysis‐dependent population is not appropriate nor feasible. The above study using cinacalcet by Mpio et al. [11] targeted patients with a PTH of > 800 pg/ml. The use of this cutoff with our patient for example would not have triggered the use of cinacalcet and his transfusion dependence would have likely persisted. Given the great morbidity associated with anemia of ESRD, and transfusion dependence in particular, providers should approach each patient with ESA‐resistant anemia as a unique case. Transfusion is associated with alloimmunization, iron overload, transfusion reactions, thrombosis and infections. Based on the findings with our patient and our institutional cohort, we suggest that it is justified to deviate from “on‐size‐fits‐all” protocols and attempt a trial of aggressive management of SHP in patients with difficult to manage ESA‐resistant anemia and more modest PTH elevation.

## AUTHOR CONTRIBUTIONS

Taha Bat, Olga K. Weinberg, Orson W. Moe and Ibrahim Ibrahim designed the study and reviewed and edited the paper. Bilal Ashraf performed the research and wrote the paper. All authors analyzed the data and approved the submission.

## CONFLICT OF INTEREST

All authors had no conflicts of interest to declare.

## References

[1] Babitt JL , Lin HY. Mechanisms of anemia in CKD. J Am Soc Nephrol. 2012;23(10):1631–4. 22935483 10.1681/ASN.2011111078 PMC3458456

[2] Fishbane S , Spinowitz B. Update on anemia in ESRD and earlier stages of CKD: core curriculum 2018. Am J Kidney Dis. 2018;71(3):423–35. 29336855 10.1053/j.ajkd.2017.09.026

[3] Kanbay M , Perazella MA , Kasapoglu B , Koroglu M , Covic A. Erythropoiesis stimulatory agent‐ resistant anemia in dialysis patients: review of causes and management. Blood Purif. 2010;29(1):1–12. 19816014 10.1159/000245041

[4] Santos EJF , Hortegal EV , Serra HO , Lages JS , Salgado‐Filho N , Dos Santos AM . Epoetin alfa resistance in hemodialysis patients with chronic kidney disease: a longitudinal study. Braz J Med Biol Res. 2018;51(7):e7288. 29742267 10.1590/1414-431X20187288 PMC5972010

[5] Zingraff J , Drueke T , Marie P , Man NK , Jungers P , Bordier P . Anemia and secondary hyperparathyroidism. Arch Intern Med. 1978;138(11):1650–2. 718313

[6] Wu SG , Jeng FR , Wei SY , Su CZ , Chung TC , Chang WJ , et al. Red blood cell osmotic fragility in chronically hemodialyzed patients. Nephron. 1998;78(1):28–32. 9453400 10.1159/000044878

[7] Rao DS , Shih MS , Mohini R. Effect of serum parathyroid hormone and bone marrow fibrosis on the response to erythropoietin in uremia. N Engl J Med. 1993;328(3):171–5. 8417383 10.1056/NEJM199301213280304

[8] Brickman AS , Sherrard DJ , Jowsey J , Singer FR , Baylink DJ , Maloney N , et al. 1,25‐dihydroxycholecalciferol. Effect on skeletal lesions and plasma parathyroid hormone levels in uremic osteodystrophy. Arch Intern Med. 1974;134(5):883–8. 4613310 10.1001/archinte.134.5.883

[9] Barbour GL. Effect of parathyroidectomy on anemia in chronic renal failure. Arch Intern Med. 1979;139(8):889–91. 464702

[10] Drueke TB , Ritz E. Treatment of secondary hyperparathyroidism in CKD patients with cinacalcet and/or vitamin D derivatives. Clin J Am Soc Nephrol. 2009;4(1):234–41. 19056615 10.2215/CJN.04520908

[11] Mpio I , Boumendjel N , Karaaslan H , Arkouche W , Lenz A , Cardozo C , et al. Secondary hyperparathyroidism and anemia. Effects of a calcimimetic on the control of anemia in chronic hemodialysed patients. Pilot Study. Nephrol Ther. 2011;7(4):229–36. 21353659 10.1016/j.nephro.2011.01.008

[12] Tanaka M , Yoshida K , Fukuma S , Ito K , Matsushita K , Fukagawa M , et al. Effects of secondary hyperparathyroidism treatment on improvement in anemia: results from the MBD‐5D study. PLoS One. 2016;11(10):e0164865. 27764168 10.1371/journal.pone.0164865 PMC5072648

